# Supplementary material for: Content-rich biological network constructed by mining PubMed abstracts
Source: BMC Bioinformatics. 2004 Oct 8;5:147. doi: 10.1186/1471-2105-5-147 (PMC528731; doi:10.1186/1471-2105-5-147)
Supplement: Additional File 2 — The original results of the above study (non-essential files are deleted to keep the file size under the limit set by BMC bioinformatics). [file 1471-2105-5-147-S2.bz2 › chilibotAdditionalFile2/dip10/89ID7962001E326/html/RECO_RECR.html]

 


 **RECO** and **RECR** 
  
Found 42 abstracts in PubMed, retrieved 10.  
 

 What does Google say? 
 PDF only 
| .edu only 

---

**Interactive relationship** (e.g. stimulation, inhibition, etc)

**Non-interactive relationship** (e.g. studied together, co-existance, homology, etc.)

- The defects in restart attributed to mutations in recF,  **recO**  or  **recR**  were suppressed in a recA730 lexA Def strain expressing constitutively activated RecA RecA\*.  Ref: 11929519 Mol Microbiol, 2002
- We analyzed effects of overexpression of RecE and RecT on illegitimate recombination during prophage induction in Escherichia coli and found that frequencies of spontaneous and UV induced illegitimate recombination are enhanced by coexpression of RecE and RecT in the wild type, but the enhanced recombination was reduced by recJ,  **recO** , or  **recR**  mutation.  Ref: 12169595 J Bacteriol, 2002
- ii In a recBC sbcA background, in which RecE mediated recombination is active, recA, recJ, recQ, recE, recT, recF,  **recO** , and  **recR**  mutations led to their accumulation.  Ref: 12718760 BMC Mol Biol< MedlineTA>BMC Mol Biol, 2002
- Genetic evidence suggests that the RecF,  **RecO** , and  **RecR**  RecFOR proteins participate in a common step of DNA recombination and repair, yet the biochemical event requiring collaboration of all three proteins is unknown.  Ref: 12769856 Mol Cell, 2003
- We compared the recombination proficiencies of recB1080,  **recO** ,  **recR** , and recF single mutants and recB1080  **recO** , recB1080  **recR** , and recB1080 recF double mutants.  Ref: 12618388 Genetics, 2003
- The results indicated that RecET mediated illegitimate recombination depends on the functions of RecJ,  **RecO** , and  **RecR** , suggesting that the RecE and RecJ exonucleases play different roles in this recombination pathway.  Ref: 12169595 J Bacteriol, 2002
- However, lexA Def  **recO**  or lexA Def  **recR**  strains, although unable to facilitate PriA pol II dependent restart, were able to perform pol V dependent readthrough.  Ref: 11929519 Mol Microbiol, 2002
- Similarly, recF,  **recO**  or  **recR**  mutations suppress the lethality of the Escherichia coli rep uvrD double mutant.  Ref: 12065426 EMBO J, 2002
- dnaC suppressors of priA overcome this inviability, especially when RecF,  **RecO**  or  **RecR**  is inactivated, indicating that RdgC avoids or counters a toxic effect of these proteins.  Ref: 12554673 EMBO J, 2003
- the  **RecO**  and  **RecR**  proteins also play important roles in the recombination.  Ref: 12169595 J Bacteriol, 2002
- **RecR**  protein, which is known to stimulate  **RecO**  protein to facilitate SSB protein displacement by RecA protein, inhibits annealing by  **RecO**  protein, suggesting that  **RecR**  protein may regulate the choice between the DNA strand invasion versus annealing pathways.  Ref: 12438681 Proc Natl Acad Sci U S A, 2002
- In a wild type, lexA background, mutations in recF,  **recO**  or  **recR**  block both pathways.  Ref: 11929519 Mol Microbiol, 2002
- To elucidate the role of several key proteins involved in this process, we have analysed the time dependence of replication restart in strains carrying a combination of mutations in lexA, recA, polB pol II, umuDC pol V, priA, dnaC, recF,  **recO**  or  **recR** .  Ref: 11929519 Mol Microbiol, 2002
- previously isolated mutations in B.subtilis recF, recL,  **recO**  and  **recR** , which belong to the same complementation group, all suppress the lethality of a pcrA mutation.  Ref: 12065426 EMBO J, 2002
- Roles of RecJ,  **RecO** , and  **RecR**  in RecET mediated illegitimate recombination in Escherichia coli.  Ref: 12169595 J Bacteriol, 2002
- Furthermore, we find that  **RecO**  and  **RecR**  significantly stimulate this recombination directed DNA replication reaction,.  Ref: 12667462 Mol Cell, 2003
- Resistance of E. coli strains to cisplatin as determined by plating experiments decreased in the following order parent PQ30 strain, polB >  **recO** ,  **recR** , recF > recB.  Ref: 12531023 DNA Repair Amst , 2002
